# Analysis of People’s Attitude Toward COVID-19 Vaccine and Its Information Sources in Thailand

**DOI:** 10.7759/cureus.22215

**Published:** 2022-02-14

**Authors:** Takeshi Yoda, Benjamas Suksatit, Masaaki Tokuda, Hironobu Katsuyama

**Affiliations:** 1 Department of Public Health, Kawasaki Medical School, Kurashiki, JPN; 2 Department of Health and Sports Science, Kawasaki University of Medical Welfare, Kurashiki, JPN; 3 Faculty of Nursing, Chiang Mai University, Chiang Mai, THA; 4 International College of Digital Innovation, Chiang Mai University, Chiang Mai, THA; 5 Center for International Research and Cooperation, Kagawa University, Takamatsu, JPN

**Keywords:** thailand, information, vaccine willingness, vaccine hesitancy, covid-19

## Abstract

Introduction

Coronavirus disease 2019 (COVID-19) vaccine hesitancy has become a global problem. Therefore, we aimed to determine the relationship between the information sources on vaccines and the willingness of people to be vaccinated in Thailand.

Methods

A sample of 500 respondents was drawn from an Internet research panel, and a questionnaire survey was administered to evaluate respondents’ willingness to be vaccinated by sex, age group, educational background, occupation, and presence of chronic diseases, as well as their information sources on COVID-19 vaccines. Descriptive statistics and logistic regression analysis were employed to assess the relationship between vaccine hesitancy/refusal and other variables.

Results

Our results demonstrated that 90.2% of the participants were either willing to vaccinate or were already vaccinated. By contrast, 6.0% were hesitant and 3.8% did not want to be vaccinated. Females, people with education less than master’s/bachelor’s degree or high school, day laborers, housewives, and unemployed were significantly related to vaccine hesitation/refusal. Furthermore, they were less likely than the vaccine willingness group to use information resources from the Ministry of Health, public health centers, or medical associations.

Conclusions

As vaccine hesitancy and refusal ratio were found to be substantially lower than in a previous study for Thailand and other countries, public authorities should poll the public to identify vaccine-hesitant populations and their reasoning and provide appropriate information directly to the general public.

## Introduction

Vaccines against coronavirus disease 2019 (COVID-19) were met with much skepticism when they were first introduced in the winter of 2020. Most of the skeptics were concerned that the time between the start of the pandemic and the development of the vaccine was too short and that the vaccine would not be effective or would cause unexpected adverse reactions [1,2]. Pfizer and Moderna have developed vaccines based on the latest technology using messenger RNA, the genetic information of SARS-CoV-2 [3]. Until now, most vaccines have been inactivated or weakened versions of the virus. Consequently, some skeptics spread rumors that the vaccination with this latest type of vaccine could trigger the extinction of the human race [4]. Compared with traditional communication channels, social media offers an unprecedented opportunity to spread anti-vaccination messages and allow communities to form around the anti-vaccine sentiment [5]. Thus, many researchers were worried about vaccine hesitation against COVID-19 [1,2,6]. However, vaccine expectations and willingness have increased as vaccines have been introduced in many countries, with a recognized preventive effect of more than 90%. In Japan, the willingness to be vaccinated before the introduction of the vaccine was 65.7% [7]; however, as the vaccination started, the willingness to be vaccinated increased and is now reported to be as high as 72.4% [8]. The vaccination coverage has reached 70% of the total population, and due to concerns over the spread of the Omicron strain, the third round of vaccination is now being administered by medical personnel.

Meanwhile, vaccination against COVID-19 was also introduced in Thailand at a relatively early stage of the pandemic outbreak. The COVID-19 vaccine administered in Thailand was manufactured by Sinovac Biotech Ltd. and the vaccination commenced in February 2021. Given its weaker efficacy against the Delta strain, since October 2021, Thailand has gradually switched to other vaccines such as those manufactured by AstraZeneca and Pfizer [9]. According to a previous study employing an Internet survey conducted between March and April 2021, in Thailand, 55.6% of respondents evinced their interest in receiving vaccination [10]. This result was somewhat lower than the results in previous studies in other countries. For example, in a survey conducted in Bangladesh from December 2020 to January 2021, 74.6% of respondents indicated they intended to be vaccinated [11], and in the results of a consolidated survey in 17 countries worldwide, 87.2% of respondents stated that they wanted to be vaccinated [12,13].

Willingness to be vaccinated against COVID-19 is influenced by information on both the vaccine and COVID-19. The change in the willingness of people in Thailand to be vaccinated is thought to be because of the outbreak of the Omicron strain and changes in the types of vaccines available [10]. Therefore, in this study, we conducted an Internet survey in December 2021 to clarify the relationship between the sources of information on vaccines and the willingness of people to be vaccinated against COVID-19 in Thailand.

## Materials and methods

We conducted this study in December 2021 in Thailand and employed the Internet research panel data from Global QiQUMO, which is operated by Cross Marketing Inc., Tokyo, Japan. More than 500,000 people were registered on this research panel in Thailand. The sample size was calculated using the following formula: n=λ2p(1-p)/d2 [14], where n is the sample size, λ is the confidence level, p is the response ratio, and d is the tolerance. We substituted λ=1.96, p=0.5, d=0.05, resulting in a minimum sample size of 384.16. We considered a larger margin to account for incomplete responses and had 500 respondents in total.

The questionnaire sought the following information: (1) sex, (2) age, (3) occupation, (4) educational background, (5) presence of chronic diseases (excluding COVID-19 infection), (6) willingness to receive COVID-19 vaccine (derived from the question: “Would you be willing to be vaccinated against COVID-19?” or “Have you already completed the COVID-19 vaccination?” with the response options being Yes, Unsure, and No), (7) reasons for the previous answer (multiple answers), and (8) information sources for COVID-19 vaccines (multiple answers). These items were referenced and arranged based on previous Japanese studies [7,15].

Descriptive statistics were used to evaluate vaccine willingness by sex, age group, occupation, educational background, and the presence of chronic diseases. The reasons for wanting and not wanting/hesitating to be vaccinated were estimated by sex. The source of COVID-19 vaccine information was assessed by vaccine willingness. The chi-square test was applied to evaluate categorical variables. We also analyzed the characteristics of the main reasons for vaccine hesitancy and refusal through logistic regression analysis, with sex, age group, occupation, educational background, and the presence of chronic diseases as independent variables. The significance level was set at <0.05. JMP Pro 14.1.0 (SAS Institute Inc., Cary, NC, USA) was used for all the analyses.

The study was approved by the Ethical Committee of the Kawasaki Medical School (Approval number: 5499-00). Implied consent was sought rather than formal written consent to ensure the anonymity of participants. The participants clicked on the “I agree” button before commencing the survey to indicate their consent.

## Results

Of the 500 participants, 246 (49.2%) were males, and the average age was 36.1 years (standard deviation was 11.6). Other socio-demographic characteristics are presented in Table 1.

**Table 1:** Characteristics of respondents

		N	%
Sex	Males	246	49.2
	Females	251	50.2
	Others	3	0.6
Age group	Under 19 years	23	4.6
	20–29 years	143	28.6
	30–39 years	143	28.6
	40–49 years	124	24.8
	50–59 years	52	10.4
	Over 60 years	15	3.0
Chronic diseases	None	342	68.4
	One or more	158	31.6
Education	Primary school	11	2.2
	Junior high school	19	3.8
	Senior high school	84	16.8
	Vocational school	87	17.4
	College/university	264	52.8
	Graduate school	35	7.0
Occupation	Self-employed	190	38.0
	Company employee	160	32.0
	Civil servant	46	9.2
	Day laborer	27	5.4
	Housewife/housekeeper	10	2.0
	Student	39	7.8
	Unemployed	15	3.0
	Others	13	2.6

Overall, 451 (90.2%) out of 500 participants stated that they were either willing to be vaccinated or were already vaccinated against COVID-19. Another 30 (6.0%) participants replied that they were not sure and 19 (3.8%) stated that they did not intend to get vaccinated. As illustrated in Table 2, we found considerable differences in the willingness to get vaccinated across gender, educational background, and occupation.

**Table 2:** Willingness to be vaccinated against COVID-19 by characteristics *Pearson's chi-square test

		Yes (%)	Unsure (%)	No (%)	p-Value*
Sex	Males	234 (68.0)	10 (18.6)	2 (13.4)	<0.01
	Females	214 (63.2)	20 (25.8)	17 (11.0)	
	Others	3 (100.0)	0 (0.0)	0 (0.0)	
Age group	Under 19 years	16 (69.6)	4 (17.4)	3 (13.0)	0.09
	20–29 years	132 (92.3)	6 (4.2)	5 (3.5)	
	30–39 years	133 (93.0)	7 (4.9)	3 (2.1)	
	40–49 years	108 (87.1)	10 (8.1)	6 (4.8)	
	50–59 years	48 (92.3)	2 (3.85)	2 (3.85)	
	Over 60 years	14 (93.3)	1 (6.7)	0 (0.0)	
Chronic diseases	None	308 (90.0)	21 (6.2)	13 (3.8)	0.98
	One or more	143 (90.5)	9 (5.7)	6 (3.8)	
Education	Primary school	6 (54.5)	3 (27.3)	2 (18.2)	<0.01
	Junior high school	13 (68.4)	4 (21.1)	2 (10.5)	
	Senior high school	79 (94.0)	3 (3.6)	2 (2.4)	
	Vocational school	79 (90.8)	6 (6.9)	2 (2.3)	
	College/university	241 (91.3)	14 (5.3)	9 (3.4)	
	Graduate school	33 (94.3)	0 (0.0)	2 (5.7)	
Occupation	Self-employed	175 (92.1)	5 (2.6)	10 (5.3)	<0.01
	Company employee	154 (96.3)	6 (3.7)	0 (0.0)	
	Civil servant	43 (93.5)	2 (4.3)	1 (2.2)	
	Day laborer	19 (70.4)	6 (22.2)	2 (7.4)	
	Housewife/housekeeper	6 (60.0)	3 (30.0)	1 (10.0)	
	Student	35 (89.7)	3 (7.7)	1 (2.6)	
	Unemployed	10 (66.7)	4 (26.7)	1 (6.6)	
	Others	9 (69.2)	1 (7.8)	3 (23.0)	

Relatively higher rates of hesitation and refusal to be vaccinated against COVID-19 were found among females (36.8%, chi-square test, p<0.01), those under 19 years of age (30.4%, p=0.09, not significant), those whose educational background was primary school or junior high school (45.5% and 31.6%, respectively, p<0.01), and day laborers, housewives, and unemployed (29.6%, 40.0%, and 33.3%, respectively, p<0.01).

**Figure 1:**
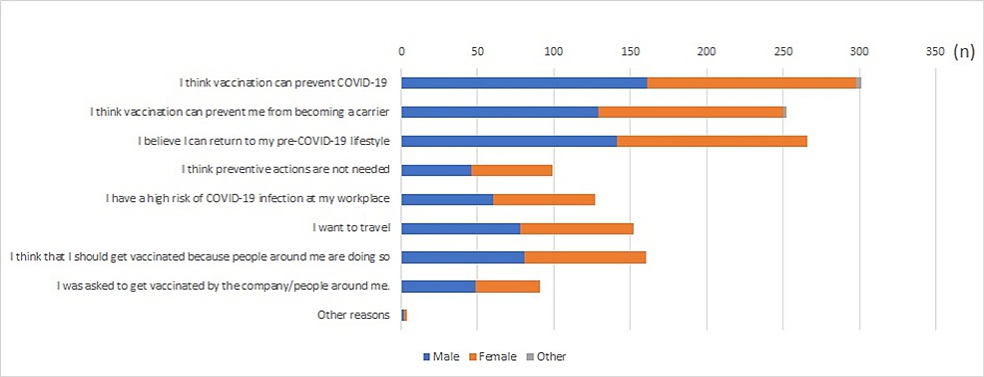
Participants’ reasons for wanting to get vaccinated or having already vaccinated against COVID-19 (n=451; multiple answers)

We asked participants who were willing to get vaccinated about their main reasons (Figure 1).

Approximately two-thirds of the participants thought that vaccination was an effective and a strong tool to prevent COVID-19 infection both for themselves (N=301, 66.7%) and for the people around them (N=252, 55.8%). However, a little over one-fifth of the participants (N=99, 21.9%) thought that after vaccination, they did not need to continue engaging in preventive measures such as social distancing and using masks.

**Figure 2:**
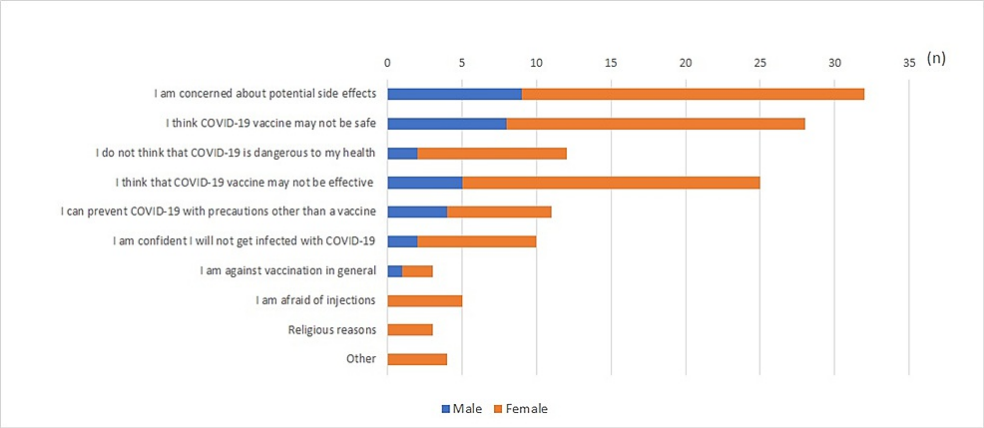
Reasons provided by participants who were either unsure or did not want to be vaccinated against COVID-19 (n=49; multiple answers)

We asked participants who were either unsure or did not want to be vaccinated against COVID-19 about the main reasons for this stance (Figure 2). Nearly two-thirds responded that they were concerned about the potential side effects of a vaccine (N=32, 65.3%). The second reason for vaccine hesitation or refusal was doubting the safety of the vaccine (N=28, 57.1%). Nearly half of the participants did not trust vaccine efficacy (N=25, 51.0%).

**Figure 3:**
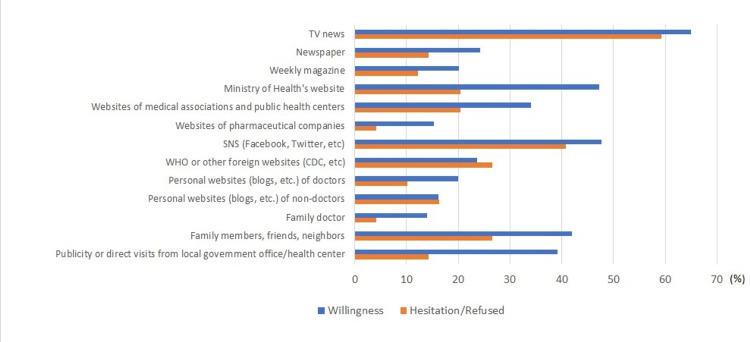
Sources of information on COVID-19 vaccine by vaccine willingness (n=451) and hesitation or refusal (n=49) Bar charts represent percentages (multiple answers) TV, television, SNS; Social Networking Service; WHO, World Health Organization; CDC, Centers for Diseases Control and Prevention

We asked participants about their sources of information on the COVID-19 vaccine (Figure 3). The sources of information between respondents who were willing to be vaccinated and those who were hesitant or refused to receive the vaccination differed greatly for the Ministry of Health’s (MOH’s) website (47.2% vs. 20.4%), the websites of medical associations and/or public health centers (34.1% vs. 20.4%), and publicity or direct visit by officers from the local government and/or public health centers (39.2% vs. 14.3%). The percentage of users of the above three information sources was higher among those who were willing to be vaccinated than among those who hesitated or refused vaccination.

Furthermore, logistic regression analyses revealed that sex (male vs. female [OR: 2.79, 95% CI: 1.25-6.22]), educational background (primary school vs. senior high school [OR: 0.13, 95% CI: 0.02-0.79]), and occupation (self-employed vs. day laborer [OR: 4.89, 95% CI: 1.53-15.56] self-employed vs. housewife/housekeeper [OR: 8.31, 95% CI: 1.87-36.88], self-employed vs. unemployed [OR: 6.35, 95% CI: 1.57-25.72]) were significant factors associated with hesitating or refusing COVID-19 vaccine (Table 3).

**Table 3:** Results of logistic regression analysis with vaccine hesitation/refusal as dependent variables *“Others” were deleted because the number was too small to analyze. Akaike’s information criterion, corrected (AICc): 295.78; Bayesian information criterion (BIC): 378.19; R^2^ = 0.206 AOR, adjusted odds ratio; CI, confidence interval

		AOR	95% CI	p-Value
Gender*	Males	1	-	
	Females	2.79	1.25–6.22	<0.01
Age group	Under 19 years	1	-	
	20–29 years	0.24	0.04–1.22	0.08
	30–39 years	0.28	0.05–1.56	0.15
	40–49 years	0.75	0.14–4.08	0.74
	50–59 years	0.38	0.05–2.77	0.34
	Over 60 years	0.47	0.03–6.93	0.58
Chronic diseases	None	1	-	
	One or more	0.86	0.41–1.84	0.71
Education	Primary school	1	-	
	Junior high school	0.70	0.10–4.79	0.72
	Senior high school	0.13	0.02–0.79	0.02
	Vocational school	0.21	0.03–1.14	0.07
	College/university	0.24	0.04–1.24	0.08
	Graduate school	0.11	0.01–1.01	0.05
Occupation	Self-employed	1	-	
	Company employee	0.46	0.16–1.28	0.13
	Civil servant	1,14	0.29–4.44	0.84
	Day laborer	4.89	1.53–15.56	<0.01
	Housewife/housekeeper	8.31	1.87–36.88	<0.01
	Student	0.73	0.14–3.70	0.70
	Unemployed	6.35	1.57–25.72	<0.01
	Others	3.87	0.88–17.04	0.07

## Discussion

In this study, an extremely large proportion of participants (90.2%) expressed their willingness to vaccinate or had already been vaccinated against COVID-19 compared with 55.6% reported in a previous study for Thailand [11], although their study period was much earlier than ours. A comparison of similar surveys in other countries reveals that the share in Japan ranged from 47% to 70% [7,8,16] and in China from 34% to 82% [17-19]. No other survey had reported that more than 90% of the respondents were willing to be vaccinated except for the USA survey [13,14]. The possible reasons for such a high rate of vaccine willingness in Thailand are as follows. First is the prevalence of the Omicron strain. The survey was conducted in the first week of December 2021, which coincides with the global outbreak of the Omicron strain [20]. Although the actual situation of this new mutant strain has not yet been clarified, it is reported to be more infectious than the delta variant [21]. This information may have increased the willingness to vaccinate. Second, the number of vaccinated people around the world has increased, leading to an increase in the availability of information on the efficacy and safety of vaccines. In particular, various studies on vaccine efficacy have demonstrated extremely high efficacy rates [3,22-24], and the increased credibility of these studies may have contributed to the higher willingness to vaccinate. Third, people felt forced by peer pressure to vaccinate. As evident in Figure 1, one-third of the participants responded, "People around me get vaccinated, so I should get vaccinated myself." Vaccination should be a decision based on one's own judgment and should not be forced. COVID-19 vaccination is not mandatory in Thailand. However, in some countries, vaccination has become compulsory [24,25], or a system has been established in which people cannot travel without vaccination [26-28]. Even if no such compulsion exists, if the majority of the people around one person are vaccinated, remaining unvaccinated will be difficult for that person as they will feel social pressure. The vaccination itself plays an important role in the prevention of COVID-19 infection; however, sometimes excessive demands for vaccination may lead to attacks on those who cannot be vaccinated because of reasons such as adverse reactions. Therefore, acting with caution is necessary.

By contrast, approximately 10% of the participants hesitated or refused to get vaccinated against COVID-19. Their main reasons for hesitation or refusal were potential side effects and safety concerns. These have also been pointed out as major reasons in previous studies [7,8,15]. The possible resolution to overcome this challenge is to properly disclose the correct information. Many researchers and government agencies (such as MOH, public health office) have shared information on adverse events as well as the efficacy of COVID-19 vaccines [29,30]. However, as illustrated in Figure 3, the vaccine hesitation/refusal group did not use the governmental information network as much as the vaccination willingness group. Even if accurate information is provided by the MOH and public health centers, it cannot be conveyed without the public accessing that information source.

Furthermore, a logistic regression analysis of factors associated with vaccine hesitancy/refusal indicated education and occupation as significant variables. The odds ratio of hesitation was higher when the educational background was elementary school compared to senior high school. The odds ratio was also significantly higher if the occupation was that of a day laborer, housewife/housekeeper, or unemployed. We also found that hesitation/refusal was higher among females than males. Our analysis of sources of information on the COVID-19 vaccine showed that people with vaccine hesitancy/refusal have less access to sources of information such as the MOH and medical associations compare with vaccine willingness. These findings suggest that the vaccine hesitancy group is not being provided with accurate information. We believe that the proactive provision of correct information not only to the public but also to these vulnerable people is necessary to overcome vaccine hesitancy.

Our study has some limitations. First, this study was Internet-based; hence, we could not eliminate the selection bias. Second, this study was a cross-sectional study, and, therefore, no causality could be established. In addition, participants may have been affected by the available information on COVID-19 at the time of the survey (first week of December 2021). The questionnaire was designed to be simple; thus, we could not evaluate other sociodemographic factors such as income, details of residence, and habits.

## Conclusions

Despite the aforementioned limitations, our study revealed that a very large number of people in Thailand are either willing to be vaccinated or have been already vaccinated against COVID-19, unlike the previous study in Thailand and other countries. Furthermore, the characteristics of people hesitating or refusing the vaccine and their sources of information were also revealed. Those who were hesitant or refused the COVID-19 vaccine were less likely to use the MOH website, governmental information such as publicities from local government offices and public health centers, and information from medical associations. Therefore, public authorities such as the MOH and public health offices should provide appropriate information directly to these vulnerable people.
